# Efficacy and safety of naotaifang capsules for hypertensive cerebral small vessel disease: Study protocol for a multicenter, randomized, double-blind, placebo-controlled clinical trial

**DOI:** 10.3389/fphar.2022.967457

**Published:** 2023-01-06

**Authors:** Rui Fang, Hua Hu, Yue Zhou, Shanshan Wang, Zhigang Mei, Ruining She, Xiwen Peng, Qiling Jiang, Xiangyuan Wang, Le Xie, Hongyuan Lin, Pan Meng, Kun Zhang, Wei Wang, Yao Xie, Litao Liu, Jiao Tong, Dahua Wu, Yunhua Luo, Chang Liu, Yifang Lu, Shangzhen Yu, Shaowu Cheng, Linyong Xu, Zhuyuan Fang, Hongcai Shang, Jinwen Ge

**Affiliations:** ^1^ School of Integrated Chinese and Western Medicine, Hunan University of Chinese Medicine, Changsha, Hunan, China; ^2^ Institute of Clinical Pharmacology of Chinese Materia Medica, Hunan Academy of Chinese Medicine, Changsha, Hunan, China; ^3^ Neurology Department, The First Hospital of Hunan University of Chinese Medicine, Changsha, Hunan, China; ^4^ School of Food and Chemical Engineering, Shaoyang University, Shaoyang, Hunan, China; ^5^ Neurology Department, Hunan Academy of Chinese Medicine Affiliated Hospital (Hunan Provincial Hospital of Integrated Chinese and Western Medicine), Changsha, Hunan, China; ^6^ Radiology Department, The First Hospital of Hunan University of Chinese Medicine, Changsha, Hunan, China; ^7^ Health Management Department, The First Hospital of Hunan University of Chinese Medicine, Changsha, Hunan, China; ^8^ Scientific Research Department, The First Affiliated Hospital of Shaoyang University, Shaoyang, Hunan, China; ^9^ Neurology Department,The First Traditional Chinese Medicine Hospital of Changde (Changde Hospital Affiliated to Hunan University of Chinese Medicine), Changde, Hunan, China; ^10^ Health Management Department, Hunan Academy of Chinese Medicine Affiliated Hospital (Hunan Provincial Hospital of Integrated Chinese and Western Medicine), Changsha, Hunan, China; ^11^ Health Management Department, The First Affiliated Hospital of Shaoyang University, Shaoyang, Hunan, China; ^12^ Neurology Department, Jiangmen Wuyi Hospital of Traditional Chinese Medicine (Jiangmen Hospital of Traditional Chinese Medicine Affiliated to Jinan University), Jiangmen, Guangdong, China; ^13^ School of Life Sciences, Central South University, Changsha, Hunan, China; ^14^ Jiangsu Province Innovation Center of TCM Hypertension Clinical Medicine, Affiliated Hospital of Nanjing University of Chinese Medicine, Najing, Jiangsu, China; ^15^ Key Laboratory of Chinese Internal Medicine of Ministry of Education and Beijing, Dongzhimen Hospital, Beijing University of Chinese Medicine, Beijing, China

**Keywords:** hypertensive cerebral small vessel disease, randomized controlled trial, Chinese medicine, naotaifang capsules, treatment

## Abstract

**Background:** Hypertensive cerebral small vessel disease (HT-CSVD) is a cerebrovascular clinical, imaging and pathological syndrome caused by hypertension (HT). The condition manifests with lesions in various vessels including intracranial small/arterioles, capillaries, and small/venules. Hypertensive cerebral small vessel disease has complex and diverse clinical manifestations. For instance, it can present as an acute stroke which progresses to cause cognitive decline, affective disorder, unstable gait, dysphagia, or abnormal urination. Moreover, hypertensive cerebral small vessel disease causes 25–30% of all cases of ischemic strokes and more than 50% of all cases of single or mixed dementias. The 1-year recurrence rate of stroke in cerebral small vessel disease patients with hypertension is 14%. In the early stage of development, the symptoms of hypertensive cerebral small vessel disease are concealed and often ignored by patients and even clinicians. Patients with an advanced hypertensive cerebral small vessel disease manifest with severe physical and mental dysfunction. Therefore, this condition has a substantial economic burden on affected families and society. Naotaifang (NTF) is potentially effective in improving microcirculation and neurofunction in patients with ischemic stroke. In this regard, this multicenter randomized controlled trial (RCT) aims to furtherly evaluate the efficacy and safety of naotaifang capsules on hypertensive cerebral small vessel disease.

**Methods:** This study is a multicenter, randomized, double-blind, placebo-controlled clinical trial. A total of 388 eligible subjects were recruited from the First Hospital of Hunan University of Chinese Medicine, Hunan Academy of Chinese Medicine Affiliated Hospital, the First Hospital of Shaoyang University, the First Traditional Chinese Medicine Hospital of Changde, and Jiangmen Wuyi Hospital of Traditional Chinese Medicine from July 2020 to April 2022. After a 4-week run-in period, all participants were divided into the intervention group (represented by Y-T, N-T) and control group (represented by Y-C, N-C); using a stratified block randomized method based on the presence or absence of brain damage symptoms in hypertensive cerebral small vessel disease (represented by Y and N). The Y-T and N-T groups were administered different doses of naotaifang capsules, whereas Y-C and N-C groups received placebo treatment. These four groups received the treatments for 6 months. The primary outcome included Fazekas scores and dilated Virchow-robin spaces (dVRS) grades on magnetic resonance imaging (MRI). The secondary outcomes included the number of lacunar infarctions (LI) and cerebral microbleeds (CMB) on magnetic resonance imaging, clinical blood pressure (BP) level, traditional Chinese medicine (TCM) syndrome scores, mini-mental state examination (MMSE) scale, and safety outcomes. Fazekas scores, dilated Virchow-robin spaces grades, and the number of lacunar infarctions and cerebral microbleeds on magnetic resonance imaging were tested before enrollment and after 6 months of treatment. The clinical blood pressure level, traditional Chinese medicine syndrome scores, mini-mental state examination scale and safety outcomes were tested before enrollment, after 3-month, 6-month treatment and 12th-month follow-up respectively.

**Conclusion:** The protocol will comfirm whether naotaifang capsules reduce Fazekas scores, dilated Virchow-robin spaces grades, and the number of lacunar infarctions and cerebral microbleeds, clinical blood pressure, increase mini-mental state examination scores, traditional Chinese medicine syndrome scores of Qi deficiency and blood stasis (QDBS), and improve the quality of life of subjects. The consolidated evidence from this study will shed light on the benefits of Chinese herbs for hypertensive cerebral small vessel disease, such as nourishing qi, promoting blood circulation and removing blood stasis, and dredging collaterals. However, additional clinical trials with large samples and long intervention periods will be required for in-depth research.

**Clinical Trial registration:** www.chictr.org.cn, identifier ChiCTR1900024524.

## Introduction

Hypertensive cerebral small vascular disease (HT-CSVD) is a series of prevalent cerebrovascular clinical, pathological, and imaging changes caused by hypertension (HT), targeting small cerebral arteries, veins, and capillaries (Liu et al., 2018). The physiological and pathological factors include cerebral arteriosclerosis, intima-media smooth muscle cell proliferation, fiberglass deposition, vascular wall thickening, lumen stenosis, or bleeding. The major clinical manifestations of HT-CSVD are cognitive impairment, abnormal gait, depression, or insomnia (Litak et al., 2020). Notably, CSVD mainly presents six typical features in magnetic resonance imaging (MRI), including recent small subcortical infarct (RSSI), vascular lacunar infarcts hyperintensity, white matter hyperintensity (WMH), dilated Virchow-robin spaces (dVRS), cerebral microbleeds (CMB) and cerebral atrophy (Wardlaw et al., 2013; Zhang et al., 2022).

In recent years, relevant studies have shown that HT-associated changes, including chronic ischemia/hypoperfusion, inflammation, oxidative stress, endothelial injury, and disruption of the blood-brain barrier (BBB) occur over time in cerebral small vessels. This potentially results in lower cognitive function when blood pressure (BP) control is poor or lacking (Litak et al., 2020). For instance, increased pulse and mean arterial pressure are conducive to forming an ischemic environment in the cerebral white matter by disrupting autoregulation and induction of endothelial cell senescence and cell death in small vessels (Kaul and Rubinstein, 2021). The above changes trigger BBB dysfunction, additional vascular permeability upregulation, and activation of pro-inflammatory mediators in localized effector cells, eventually causing substantial damage and WMH on brain MRI (Kaul and Rubinstein, 2021). Angiotensin II (Ang II) is a vasoactive peptide of the renin-angiotensin system (RAS) and an essential molecular signal in HT. Ang II induces the release of reactive oxygen species (ROS) and upregulates NADPH oxidase 2 (NOX2), NOX4 and cyclooxygenase 2 (COX2), resulting in oxidative stress and cerebrovascular dysfunction, including BBB leakage, CSVD, and cognitive impairment (Lu et al., 2021).

The onset of HT-CSVD is difficult to be diagnosed and can easily be ignored by clinicians and patients. Without effective intervention, HT-CSVD gradually progresses to cerebral hemorrhage, cerebral infarction, and other cerebral macrovascular diseases. Therefore, early intervention and treatment may be of great significance. At present, specific treatment for HT-CSVD is lacking. The current therapeutic strategies include symptomatic treatment and controlling risk factors, i.e., reducing BP, antiplatelet therapy, thrombolysis, and statin therapy (Cannistraro et al., 2019). Nevertheless, these therapies have contraindications and complications. For example, although statin therapy has demonstrated a significant beneficial effect in patients with ischemic stroke, patients receiving high-dose atorvastatin exhibit an increased risk of intracerebral hemorrhage (ICH) recurrence, specifically in patients with CSVD (Amarenco et al., 2009; Cannistraro et al., 2019). Therefore, there is an urgent need to identify new, safe and effective treatment strategies for HT-CSVD.

Traditional Chinese medicine (TCM), with the advantages of early conditioning and multi-target, is responsible for the prevention and treatment of HT-CSVD (Lin et al., 2021; Li et al., 2020; Zhou et al., 2022). Based on TCM theory, Qi deficiency and blood stasis (QDBS) is the major mechanism of HT-CSVD (Lin et al., 2021) and cerebral ischemia or ischemic/reperfusion injury. Notably, QDBS is a complex pathological system accompanied by oxygen radical accumulation, inflammation, vascular endothelial damage, and viscera injury, which in turn further promotes the development of QDBS (Bi et al., 2019; Zhang et al., 2020; Liu et al., 2021). According to TCM theory, Qi pushes the blood to circulate in the channels and can produce blood. Qi deficiency reduces blood production and cannot nourish the meridians, hence a lack of nutrition in the brain tissue. As a result, the body becomes prone to cognitive impairment and memory loss. Moreover, Qi deficiency hinders blood circulation; hence blood stasis stagnates in the veins or circulates outside the veins. The above changes in blood circulation induce cerebral vascular embolisms or CMB (Wang et al., 2021), gradually causing HT-CSVD.

The Chinese medicine Naotaifang (NTF) is the addition and subtraction of the classic formula--Buyang Huanwu Decoction, composed of four herbs: *Astragalus* mongholicus Bunge (Fabaceae) (Huangqi) 40 g, *Conioselinum anthriscoides* ‘*Chuanxiong*’ (Apiaceae) (Chuanxiong) 15 g, Pheretima aspergillum (Dilong) 15 g, and *Bombyx* Batryticatus (Jiangcan) 10 g. Single herbal medical effect and compound clinical efficacy show that NTF nourishes Qi, activates blood circulation, removes blood stasis, dredging channels, and collaterals (He et al., 2001; Lan et al., 2020; Yang et al., 2021; Xu et al., 2022). Our previous studies indicate that NTF alleviates nerve damage and improves cognitive function by suppressing neuronal cell ferroptosis (Lan et al., 2020). This reduces the infarct volume and apoptotic neuronal cells as well as increases the density of dendritic spines and survival of neurons (Yang et al., 2021). One pharmacological study reports that the metabolic pathway regulated by NTF is mainly purine metabolism. Moreover, NTF has 21 serum differential metabolites, such as adenosine, inosine, ferulic acid, calycosin, *etc.* (Xu et al., 2022). Elsewhere, another clinical study shows that NTF has a definite clinical effect (including significantly reducing neurological deficit scores, and improving hemorheology index) on cerebral infarction patients with QDBS syndrome, without obvious adverse events (AEs) and other abnormal safety outcomes (He et al., 2001; Lan et al., 2020). Based on the above studies, we reasonably speculate that NTF can prevent the progression of HT-CSVD as well as promote angiogenesis and neuroprotection. This randomized controlled trial (RCT) will provide detailed information for researchers about the study following the Standard Protocol Items: Recommendations for Interventional Trials-Traditional Chinese Medicine (SPIRIT-TCM) Extension 2018 Statement (Cheng et al., 2017; Dai et al., 2019). In strict compliance with the trial procedures and quality control, this study aims to provide high-quality evidence for the assessment of efficacy and safety of NTF capsules in treating of HT-CSVD with QDBS syndrome.

## Methods and analysis

### Study design

**FIGURE 1:**
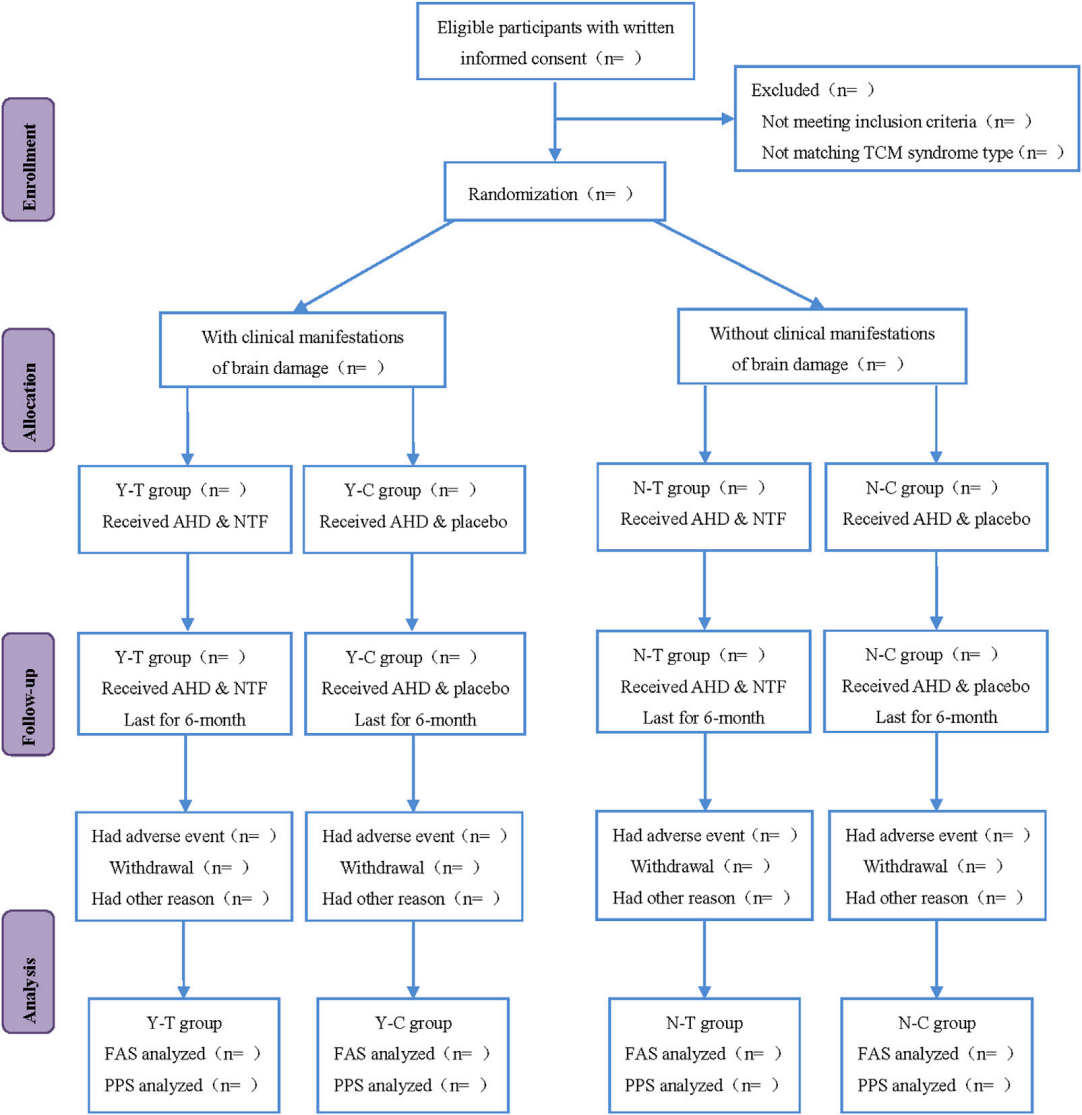
Flow chart.

**FIGURE 2:**
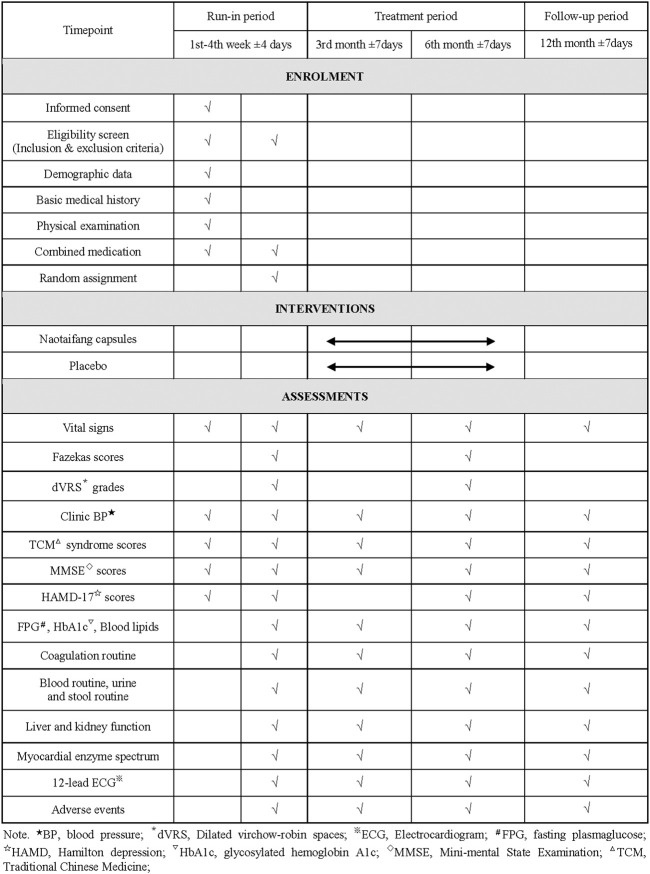
The schedule of enrollment, interventions, and assessments.

This study is a multicenter, randomized, double-blind, placebo-controlled clinical trial that will last for 13 months, including 4 weeks of the run-in period, 6 months of treatment period, and the 12th month follow-up period. Participants will be first recruited for a baseline screening, evaluation and washing-out the drug whose efficacy is similar to that of the investigational products (IP) (such as the Chinese medicine for nourishing Qi and activating blood circulation) in the run-in period. Eligible participants will be randomly classified into the intervention group and control group (all participants will be visited once after the 3rd and 6th month of treatment, respectively). Participants will be assessed with a relevant scale or laboratory biochemical examination on the scheduled day for each treatment and follow-up period. The flowchart and schedule of the trial, including a schedule of enrollment, interventions, and assessments are shown in Figures 1, 2. The trial protocol is written as per the SPIRIT-TCM Extension 2018 Statement checklist. The structured study summary is described in Table 1 according to the World Health Organization Trial Registration Dataset.

**TABLE 1:** WHO trial registration data set—structured summary.

Data category	Information
Primary registry, trial identifying number	www.chictr.org.cn (ChiCTR1900024524)
Date of registration in primary registry	13 July 2019
Sources of monetary support	The National Key Research and Development Program Project of the Ministry of Science and Technology
Contact for public queries	Rui Fang, MD (fangruitcm@126.com)
Contact for scientific queries	Jinwen Ge, Pro (001267@hnucm.edu.cn)
Public title	Evidence-based optimization study on the prevention and treatment of hypertensive cerebral small vessel disease with Naotaifang
Scientific title	A randomized, double-blind, placebo-controlled, multi-center clinical trial of Naotaifang capsules for the prevention and treatment of hypertensive cerebral small vessel disease (QDBS syndrome)
Country of recruitment	Hunan and Guangdong province, China
Health problem	Hypertensive cerebral small vessel disease (QDBS syndrome)
Intervention(s)	Intervention groups: Y-T group (4 capsules thrice daily for 24 weeks), N-T group (4 capsules twice daily for 24 weeks)
	Control groups: Y-C group and N-C group (Placebo capsules, The dose and usage will be the same as in the intervention groups)
Key inclusion and exclusion criteria	Inclusion criteria: (1) aged 40–75 years; (2) diagnosed with HT and CSVD; (3) TCM syndrome differentiation is Qi deficiency and blood stasis (QDBS), and the score of QDBS ≥ 5; (4) taking basic AHD, including calcium channel blockers (CCB), angiotensin converting enzyme inhibitors (ACEI) or angiotensin receptor blockers (ARB); (5) voluntarily signed informed consent form (ICF) to participate in this trial
	Exclusion criteria: (1) severe cardiovascular and cerebrovascular diseases (i.e., myocardial infarction, heart failure, severe arrhythmias, cerebral hemorrhage) and patients with lesions in the large blood vessels of the brain; (2) combined with severe liver, kidney, digestive system or hematopoietic system diseases; (3) uncontrolled BP after taking AHD (systolic BP
	≥180 mmHg and/or diastolic BP ≥ 110 mmHg); (4) combined with diabetes but fasting plasma glucose (FPG)>7 mol/L or random blood glucose>11.1 mol/L; (5) combined with moderate to severe dementia or depression, or other mental illness; (6) pregnant or lactating; (7) suspected or confirmed history of alcohol or drug abuse; (8) allergic reaction to the investigational products (IP); (9) have the potential risk to fall out of the group, such as frequent changes in working location, unstable living place, *etc.* (10) having participated in another clinical study within 3 months; (11) CSVD due to other diseases
Study type	Interventional allocation: randomized Intervention model: parallel assignment Masking: double blind
	Primary purpose: Treatment
	Phase II
Date of first enrollment	July 2020
Target sample size	388
Recruitment status	Completed
Primary outcome	The Fazekas scores and dVRS grades before enrollment and 6 months after completion of treatment
Secondary and safety outcomes	Secondary outcomes: the number of LI and CMB, Clinic BP, FPG, HbA1c, blood lipids (TC, HDL-C, LDL-C, TG, ApoA1, ApoB), coagulation routine (PT, APTT, FIB, TT), TCM syndrome scores, MMSE and HAMD-17 scores
	Safety outcomes: blood routine (RBC, WBC, HGB, PLT), urine routine (WBC, RBC, urine glucose), stool routine (qualitative + occult blood), liver function (ALT, AST, GGT, TBIL, ALP), renal function (BUN, Cr, UA, eGFR), myocardial enzyme spectrum (CK, CK-MB, LDH), and ECG before enrollment, after 3 and 6 months of treatment

### Participants

Participants will be recruited and screened in the following five clinical research sub-centers: the First Hospital of Hunan University of Chinese Medicine, Hunan Academy of Chinese Medicine Affiliated Hospital, Jiangmen Wuyi Hospital of Traditional Chinese Medicine, the First Traditional Chinese Medicine of Changde, and the First Affiliated Hospital of Shaoyang University. The trial covers the period from July 2020 to April 2022.

### Recruitment

Most participants will be recruited through recruitment advertisements and posters in the five selected clinical research sub-centers. Other participants diagnosed with HT will be invited through the research team or clinical observers by telephone. All participants must complete a brain MRI examination to confirm the diagnosis of CSVD and be re-screened by a neurologist. All participants will undergo a standardized interview and receive clinical study information concerning the trial. Each participant should sign an informed consent form (ICF).

### Diagnostic criteria

#### Diagnostic criteria of hypertensive

According to the “2018 Chinese guidelines for the management of Hypertension” (Writing Group of 2018 Chinese Guidelines for the Management of Hypertension, 2019), in the absence of anti-hypertensive drugs (AHD), clinical BP is measured three times on different days, with systolic BP not lower than 140 mmHg and/or diastolic BP not lower than 90 mmHg, or those who have been diagnosed with HT by a specialist in the past and are currently taking AHD.

#### Diagnostic criteria of cerebral small vascular disease

**FIGURE 3:**
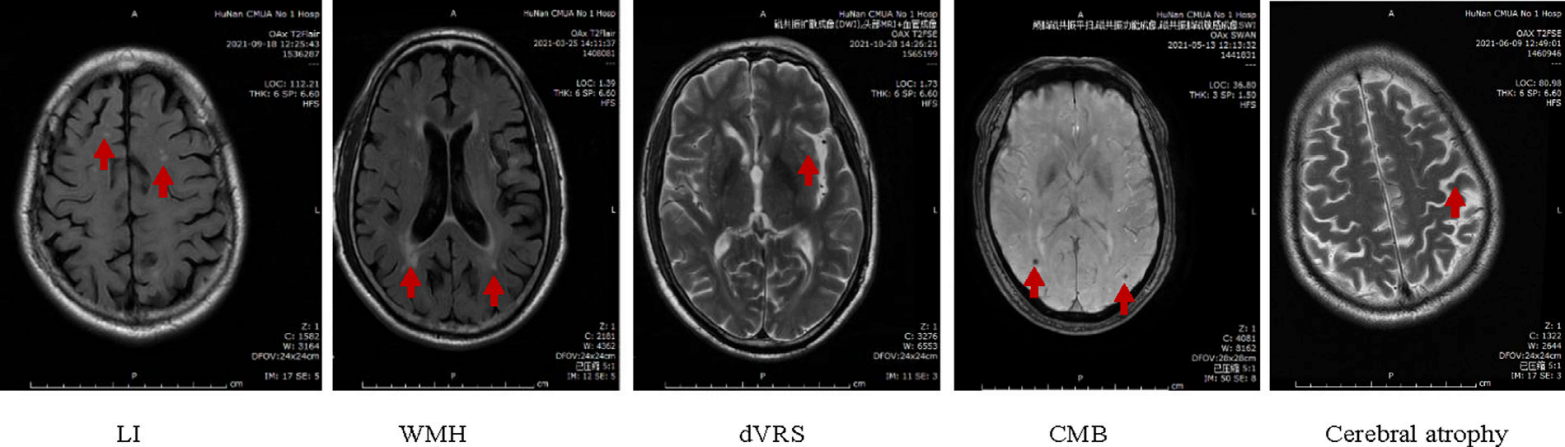
Imaging features of CSVD.

According to the “Chinese Consensus on Diagnosis and Therapy of CSVD” (Huang, 2015) and Standards for ReportIng Vascular changes on nEuroimaging (STRIVE v1) (Wardlaw et al., 2013), CSVD is primarily characterized by stroke (small deep infarction and cerebral hemorrhage), cognitive and emotional impairment and overall functional decline. Based on brain MRI, prominent manifestations include lacunar infarction (LI), WMH, dVRS, CMB, and cerebral atrophy (Figure 3). The imaging features include:

(1) Number of LI: (a) Old LI: round or oval, with a maximum diameter not exceeding 15mm, distributed under the cortex, T1 and T2 are cerebrospinal fluid signals and T2 fluid-attenuated inversion recovery (FLAIR) is a ring of low signal in the center and high signal around it; (b) Acute LI: round or oval, with a maximum diameter not exceeding 15 mm, subcortical distribution, high signal on diffusion-weighted imaging (DWI) and negative on conventional MRI.
(2) WMH of vascular origin: It shows abnormal signals, and different ranges and sizes in the white matter. Such as an equal signal or low signal in T1, a high signal in T2, or T2 FLAIR without a cavity. The severity of cerebral white matter lesion (WML) is based on the Fazekas scores (Fazekas et al., 1987); for instance, changes in paraventricular hyperintensity, such as no lesions, cap-like or pencil-thin lesions, smooth halo, irregular paraventricular hyperintensity, extend to the deep white matter, rated as 0, 1, 2, and 3 points respectively; the signal changes in the deep white matter, such as no lesions, spot-like or small-piece lesions, extensive patchy lesions beginning to fuse and large-area fusion of lesions, score 0, 1, 2 and 3 respectively. The total scores are 0–6 points. WMH caused by other non vascular diseases should be excluded, such as multiple sclerosis, white matter dystrophy, etc.
(3) dVRS grades (Doubal et al., 2010): The gap that passes through the gray or white matter and is consistent with the course of blood vessels. Round or oval or linear, with smooth margins, maximum diameter exceeds 3 mm, located in the distribution area of perforating arteries, all sequences are consistent with cerebrospinal fluid signal, T2 FLAIR has no surrounding hyperintensity ring (except when it crosses the WMH area), DWI has no diffusion restriction. (a) In the basal ganglia, the level with the most dVRSs prevails, and the severity is rated as 4. The classification is as follows: Grade 0 = no dVRS, Grade 1 ≤ 5 dVRS, Grade 2 = 5–10 dVRS, Grade 3 ≥ 10 dVRS, but the number of dVRS is still countable. The number of dVRS Grade 4 is difficult to count, and Grade 4 has cribriform changes in the basal ganglia. (b) In the white matter area, dVRS severity scoring criteria: Grade 0 = no dVRS, Grade 1 ≤ 10 dVRS, Grade 2 ≥ 10 dVRS (but the largest level of dVRS is < 10), Grade 3 = 10–20 dVRS at the maximum number level and Grade 4 ≥ 20 dVRS at the maximum number level.
(4) CMB: Round or oval, well-defined, homogeneous susceptibility-weighted imaging (SWI) signal focal [computed tomography (CT) is not shown in T1, T2, T2 FLAIR], the diameter is 2–10 mm, and the lesion is surrounded by brain parenchyma. A hypointense signal <2 mm seen on 1.5T MR may only be the missing signal in one voxel, and the artifact cannot be ruled out.
(5) Cerebral atrophy: Reduction of brain volume unrelated to specific focal injury.

If either (1) or (2) is positive, it means that the patient is diagnosed as CSVD, which may be accompanied by dVRS, CMB, cerebral atrophy, etc.

To ensure the consistency of acquisition parameters in different centers, neurology and imaging experts will formulate unified evaluation criteria for imaging parameters as per the latest guidelines. Moreover, standardized training for imaging physicians in each sub-center will be conducted before the start of the trial.

### Inclusion and exclusion criteria

#### Inclusion criteria

Participants who meet the following criteria will be included in the trial:

(1) Aged 40–75 years;
(2) Diagnosed with HT and CSVD;
(3) TCM syndrome differentiation is QDBS, and the score of QDBS ≥ 5;
(4) Taking basic AHD, including calcium channel blockers (CCB), angiotensin converting enzyme inhibitors (ACEI), or angiotensin receptor blockers (ARB);
(5) Voluntarily signed ICF to participate in this trial.

#### Exclusion criteria

Participants who meet the following criteria will be excluded from the trial:

(1) Severe cardiovascular and cerebrovascular diseases (i.e., myocardial infarction, heart failure, severe arrhythmias, cerebral hemorrhage) and patients with lesions in the large blood vessels of the brain;
(2) Combined with severe liver, kidney, and digestive system or hematopoietic system diseases;
(3) Uncontrolled BP after taking AHD (systolic BP ≥ 180 mmHg and/or diastolic BP ≥ 110 mmHg);
(4) Combined with diabetes but fasting blood-glucose >7 mol/L or random blood glucose >11.1 mol/L;
(5) Combined with moderate to severe dementia or depression, or other mental illness;
(6) Pregnant or lactating;
(7) Suspected or confirmed history of alcohol or drug abuse;
(8) Allergic reaction to the IP;
(9) Have the potential risk to fall out of the group, such as frequent changes in working location, unstable living place, *etc.*;
(10) Having participated in another clinical study within 3 months;
(11) CSVD due to other diseases.

### Preparation, components analysis and identification of naotaifang capsules and placebo

The preparation and production of NTF capsules were conducted as follows: Every NTF capsule contains 4 Chinese herbs: Astragalus mongholicus Bunge [Fabaceae; Roots] (Huangqi, 0.665 g), Conioselinum anthriscoides “Chuanxiong” [Apiaceae; Rhizomes] (Chuanxiong, 0.25 g), Pheretima aspergillum [Megascolecidae; Bodies] (Dilong, 0.25 g) and Bombyx Batryticatus [Bombycidae; Bodies] (Jiangcan, 0.165 g), with a specification of 0.267 g infused powder per capsule (approximately 1.33 g of NTF raw herb). These four Chinese herbs were mixed according to the proportion of prescription, then 8 times the amount of purified water was added. The pressure was increased and Chinese herbs were extracted twice, each time for 1.0 h. It was then filtered and the resultant filtrates from the two steps were combined to form the total filtrate. At 70–80°C temperature, the total filtrate was concentrated and dried into dry paste powder under reduced pressure. Dextrin (calculated as per the formula [dextrin amount = 60 pills × 0.4 g/pill – the amount of dry paste powder – the amount of magnesium stearate]) was added to the mixture. Soft materials were prepared through the wet method with an appropriate volume of 90-95% ethanol. The soft materials were sifted into wet particles using an 18–20 mesh sieve which were then dried at 50–70°C. Subsequently, the dry particles were sieved to remove fine particles through an 18–20 mesh sieve. Finally, a small amount of magnesium stearate (0.5%) was added and mixed evenly, the mixture was placed into the gelatine hollow capsules.

Placebo was developed based on caramel and dextrin, with appropriate taste regulation agents, including magnesium stearate, color regulates agents, and excipients, whose package, color, and the flavor was consistent with that of NTF capsules.

Thin-layer chromatography (TLC) and high-performance liquid chromatography (HPLC) were used to characterize the primary active ingredients of NTF capsules. Astragaloside A and ferulic acid (the main active ingredient in *Astragalus* mongholicus Bunge [Fabaceae] and Conioselinum anthriscoides “Chuanxiong” [Apiaceae]) were selected as the quality control indicators for content determination. The two active ingredients were consistent with the control index of *Astragalus* mongholicus Bunge [Fabaceae] and Conioselinum anthriscoides “Chuanxiong” [Apiaceae] contents in NTF capsules, and can trace the value transmission process to a certain extent. After producing the NTF capsules, each test item conformed to the requirements of the quality standard, and validated the identification and content results, all of which met the verification requirements.

**FIGURE 4:**
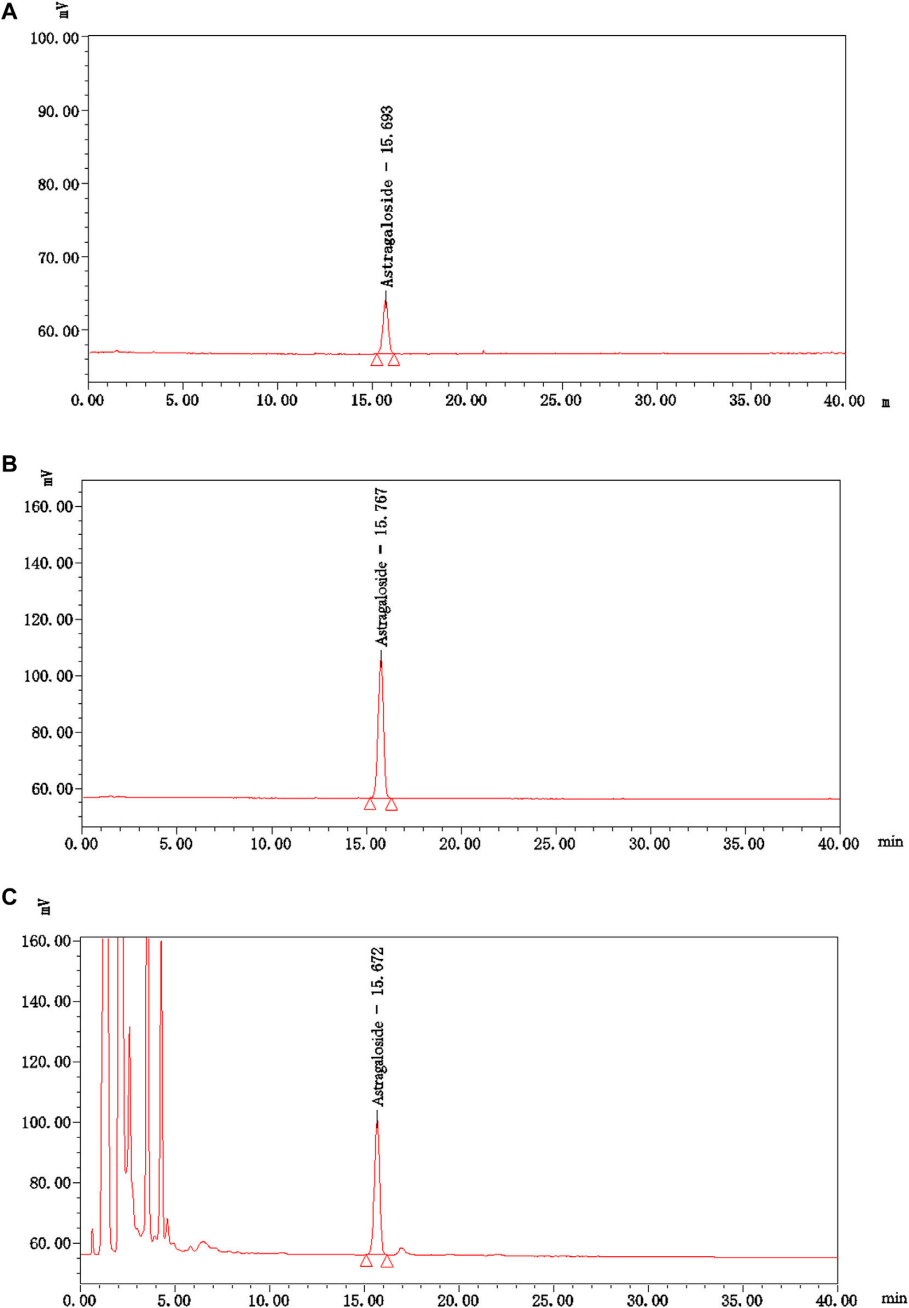
The HPLC chromatograms of astragaloside A and NTF capsules. **(A)** The control sample (astragaloside A) solution-10 μL; **(B)** The control sample (astragaloside A) solution-30 μL; **(C)** The test sample (NTF capsules) solution-20 μL.

The content of astragaloside A (C_41_H_68_O_14_), i.e., the main active ingredient of *Astragalus* mongholicus Bunge [Fabaceae] in NTF capsules was 0.44 mg/g, and the specific analysis method was: the chromatographic column of Apollo C18 (4.6 × 250 mm, 5 μm), column temperature of 40°C; Acetonitrile water (32:68) was used as the mobile phase with a flow rate of 1.0 mL/min; The evaporative light scattering detector (Altay ELSD6000, drift tube temperature of 105°C, gas flow of 2.5 L/min) was used for detection. Subsequently, 10 μL and 30 μL of control sample (astragaloside A) solution and 20 μL of test sample (NTF capsules) solution were collected and injected into the HPLC for measurement, respectively. The Empower 3 software was used to collect and analyze data with an external standard two-point logarithmic equation (Figure 4).

Preparation of control sample solution: An appropriate amount of the control sample-astragaloside A was accurately weighed, and methanol was added to prepare a solution containing 0.06 mg per 1.0 mL. Preparation of test sample solution: Approximately 1.0 g of the test sample-NTF capsules was weighed, mixed in 20 mL of water, shaken, and extracted 4 times using n-butanol mixed with the saturating water, 20 mL each time. The extracts from the 4 rounds were combined, washed twice with the ammonia test solution, 10 mL each time, and then washed twice with the water saturated mixture of n-butanol, 10 mL each time. It was then evaporated to dryness with n-butanol solution, the residue was dissolved in methanol, and transferred to a 5 mL-volumetric flask. Finally, methanol was added to the scale, shaken, and filtered to collect the filtrate.

**FIGURE 5:**
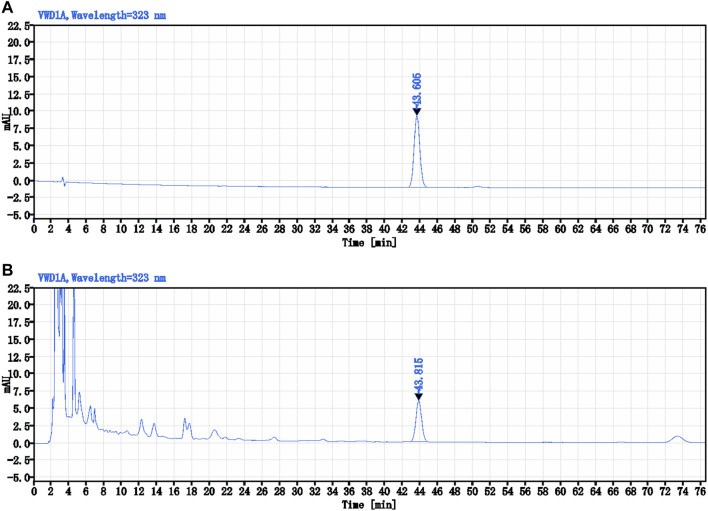
The HPLC chromatograms of Ferulic acid and NTF capsules. **(A)** The control sample (Ferulic acid) solution-10 μL; **(B)** The test sample (NTF capsules) solution-10 μL.

Ferulic acid (C_10_H_10_O_4_), i.e., the main active ingredient of Conioselinum anthriscoides “Chuanxiong” [Apiaceae] in NTF capsules was 0.21 mg/g. The specific analysis method was as follows: CAPCELL PAK C18 TYPE MG (4.6 × 250 mm, 5 μm), column temperature 30°C; Methanol – 2% acetic acid solution (18:82) was used as mobile phase at a flow rate of 1.0 mL/min. The ultraviolet detector was used to record at a wavelength of 323 nm. 10 μL of the control product (ferulic acid) solution and 10 μL of test product (NTF capsules) were injected into the HPLC for measurement, respectively. The chemstation software was used to collect and calculate data based on the external standard method (Figure 5).

Preparation of control sample solution: An appropriate amount of control sample-ferulic acid was weighed and placed in a brown measuring flask. A concentration of 45% ethanol glacial acetic acid (20:1) solution was added to prepare a solution containing 10 μg per 1.0 mL. Preparation of test sample solution: Exactly 1.0 g of the test sample - NTF capsules content was weighed and placed in a conical flask with a stopper. We then added 40 mL of 45% ethanol glacial acetic acid (20:1) solution into the flask, sealed it tightly, heated, and refluxed for 1.0 h after weighing. It was then cooled and weighed again. Next, 45% ethanol glacial acetic acid (20:1) solution was added to make up for the lost weight, vigorously shaken, and centrifuged. The supernatant was filtered to obtain a clean sample.

Hinye Pharmaceutical Co., Ltd. manufactured NTF capsules and placebo. According to the Chinese Pharmacopoeia (2015) (Chinese Pharmacopoeia Commission, 2015), the entire production process of IP strictly adheres to good manufacturing practice (GMP) standards. Besides, the production process conforms to the quality specification standards. The quality-checked IP will be delivered to each clinical research sub-center. The medication administrator in every research sub-center receives and manages drugs following good clinical practice (GCP) requirements. Storage conditions: room temperature within 25 ± 2°C and relative humidity range of 60 ± 10%.

### Group assignment and intervention

Participants will be stratified and denoted by Y or N according to the presence or absence of clinical manifestations of brain damage (cognitive decline, mood disorders, swallowing disorders, unstable gait, prolonged dizziness). Y and N groups will be divided into intervention groups (denoted by Y-T and N-T) and control groups (denoted by Y-C and N-C) using the stratified block randomized method with SAS statistical software.

Different doses of NTF capsules will be given to the Y-T and N-T groups, i.e., the Y-T group will be administered 4 capsules thrice daily with warm water, half an hour after breakfast, lunch, and dinner. In contrast, the N-T group will be given 4 capsules twice daily with warm water, half an hour after breakfast and dinner. The Y-C and N-C groups will receive the placebo. The dose and usage of the placebo in the Y-C and N-C groups will be similar to that in the intervention groups. The treatment for the four groups will last 6 months.

In each center, every participant will be assigned a clinical research coordinator (CRC) responsible for allocating drugs based on whether they have clinical manifestations of brain damage and recruitment sequence before filling out the dispensing record form.

### Randomization

This study uses a stratified block randomization approach. The participants were grouped into HT-CSVD with clinical manifestations of brain damage and HT-CSVD without clinical manifestation of brain damage based on the presence or absence of clinical manifestations of brain damage. An appropriate block length was selected (the size of block is 4) and random assignment was conducted using the SAS 9.4 statistical software (SAS Institute, North Carolina, United States) developed by statisticians from the Drug Clinical Evaluation Research Center of Central South University. 194 random sequences were generated for each group, that is, the randomized coding table with No. 001-194 was developed. The participants of each group were randomly divided into the intervention group and the control/placebo group at an allocation ratio of 1:1. Randomized codes were used as the drug numbers which were kept in an opaque sealed envelope and taken to Hinye Pharmaceutical Co., Ltd. (Changsha Hunan, China) for unified drug label production. The identification code of each subject was similar to the drug number.

### Blinding

The trial is a double-blinded design in which all the investigators, clinical observers, and participants are blinded to treatment assignments. Randomized codes will be completed by the statisticans. The allocation of unique codes for each group will be managed by the staff of Hinye Pharmaceutical Co., Ltd.

The record of the blinding process will be kept as one of the clinical trial documents. The contents of the blinding record include drug preparation, drug packaging, usage, storage and assignment, generation of randomized codes, drug box production, emergency letter, test report of NTF capsules and placebo, the storage of blinding codes, rules for breaking blind law and dispensing numbers of each research center.

Blinded coding, initial randomization number, block length, and other parameters are sealed into two copies. For proper storage, the two copies were handed to the leader of the five clinical research sub-centers and trial sponsor of the study at the Department of Drug Clinical Trial Administration in the First Hospital of Hunan University of Chinese Medicine, and Hunan University of Chinese Medicine, respectively. The blinding codes will be kept strictly confidential by an impartial third party and remain sealed until statistical analysis at the end of the trial. The assignment codes will be opened only in case of a AEs relevant to the IP and other emergencies.

### Data collection

#### Baseline data

Baseline data, including general demographic information, past medical history, symptoms and signs, history and treatment of HT, brain MRI, TCM syndrome scores, cognitive and depression scales and safety evaluations will be collected. The content includes the subject’s Age, gender, occupation, body mass index (BMI) and clinical BP, main symptoms and signs, history of basic diseases and medication, TCM syndrome scores, mini-mental state examination (MMSE) and Hamilton depression (HAMD)-17 scores, brain MRI (Fazekas scores, dVRS grades, number of LI and CMB), electrocardiograph (ECG) and laboratory parameters including blood routine, urine and stool routine, liver and kidney function, blood lipids, fasting plasma glucose (FPG), glycosylated hemoglobin A1c (HbA1c), myocardial enzyme spectrum and coagulation routine.

#### Assessment data

After 3 and 6 months of treatment, the subjects’ clinical BP, main symptoms and signs, disease history and medication use, TCM syndrome scores, MMSE and HAMD-17 scores will be assessed. Laboratory examination results will be collected respectively, such as blood routine, urine and stool routine, liver and kidney function, blood lipids, FPG, HbA1c, myocardial enzyme spectrum and coagulation routine, and ECG. The onset time, cause, and treatment of AEs will be recorded in the case report form (CRF), including an analysis of whether AEs are associated with the IP, completion and suspension time of the trial. Brain MRI will be assessed after 6 months of treatment (included Fazekas scores, dVRS grades, number of LI and CMB).

### Indicators detection

The clinical BP will be measured at 8–9 am in a fasting state using an upper arm electronic sphygmomanometer, such as TM-2655P, TM-2656VP (A&D Company, Ltd., Tokyo, Japan) or HEM-7124(Omron Company, Ltd., Dalian, China). The subjects should rest for 10–15 min before BP measurement, then take a sitting position with the palm stretched upwards, the elbow at the heart level, the upper arm at 45° to the torso, and the hand relaxed without clenching the fist. The patient should wait for a while if any abnormality is found during the BP measurement. The interval between two measurements should not be less than 5 min, the site and position of the measurement should be similar. Brain MRI will be performed using the US GE SignaHDxt 3.0T or 1.5T.

During the run-in period and the treatment periods, 3–5 ml of peripheral venous blood will be drawn from all subjects in the morning on an empty stomach, then left to stand for 1–2 h. Whole blood, serum, and plasma supernatant (extracted after centrifugation at 3,000 rpm for 10–15 min) will be taken for laboratory biochemical examination, including blood routine, myocardial enzyme spectrum, liver and kidney function, blood lipids, FPG, and HbA1c. Meanwhile, three tubes of midstream specimen of urine from each subject will be taken for urine sediment microscopy, urine routine, and microalbumin analysis. Appropriate stool samples will be kept for stool routine and occult blood tests. Laboratory biochemical examination will be performed using the cobas8000 (702/502), cobas U601/701 Roche automatic biochemical analyzer, and SYSMEX-Xi9000 hematology analyzer.

### Types of outcomes

#### Primary outcome

The Fazekas scores and dVRS grades will be obtained for both groups. The specific scoring criteria are described in the diagnostic criteria. Brain MRI examinations will be performed before enrollment and after 6 months of treatment.

#### Secondary outcomes

The number of LI and CMB will be examined using brain MRI before enrollment and after 6 months of treatment (specific scoring criteria are described in diagnostic criteria). Clinical BP, FPG, HbA1c, blood lipids (TC, HDL-C, LDL C, TG, ApoA1, ApoB), coagulation routine (PT, APTT, FIB, TT), TCM syndrome scores, and MMSE scores will be collected before enrollment and after 3 and 6 months of treatment, respectively. TCM syndrome scores will be obtained for cardinal symptoms of hard breath or fatigue, pain, amnesia, hemiplegia, limb numbness and dizziness (None = 0, Mild = 2, Moderate = 4, Severe = 6), and minor symptoms of spontaneous sweating, lazy speech, phlegm and mania (None = 0, Mild = 1, Moderate = 2, Severe = 3), with total scores of 0–48 (Zhen, 2002). The maximum scores are 30 in MMSE scale. Scores of 27–30 are considered normal, whereas scores <27 are considered a cognitive impairment. Dementia severity grading: The total scores of MMSE scale≥ 21 for mild, 10–20 for moderate, and ≤9 for severe (Folstein et al., 1975).

#### Safety outcomes

The safety outcomes will include any AEs and clinically meaningful changes in vital signs or laboratory examination during the trial, including blood routine (RBC, WBC, HGB, PLT), urine routine (WBC, RBC, urine glucose), stool routine (qualitative + occult blood), liver function (ALT, AST, GGT, TBIL, ALP), renal function (BUN, Cr, UA, eGFR), myocardial enzyme spectrum (CK, CK-MB, LDH), and ECG. These evaluations will be performed before enrollment and after 3, 6-month treatment and 12-month follow-up respectively.

### Quality control and data monitoring

First, all investigators will receive unified training according to the investigator’s instruction manual before the start of the study. The principal investigator will convene monthly meetings to discuss and resolve the problems in the trial (including subject recruitment, withdrawal, AEs, data collection and protocol modifications). Additionally, investigators shall obtain ICF from the trial subjects.

Secondly, CRF is filled by investigators and CRC according to the investigator's brochure in every clinical research sub-center. Subjects with basic diseases, including stroke and coronary heart disease, who have been taking medication for a long time and are stable, should continue to participate in the trial, however, combined drugs must be filled in the CRF. Those with unstable basic diseases will be excluded from the study. Subjects experiencing allergies or other side-effects or intolerance during medication or aggravated due to basic diseases and those unwilling to continue to participate for some reasons such as the COVID-19 pandemic should record their reasons for withdrawal in the CRF. In case of an emergency, and there is a need to uncover the blinding codes, the date and reason shall be filled in CRF.

Thirdly, regarding data monitoring and management, Clinical research associate (CRA) from Hinye Pharmaceutical Co., Ltd. will check the CRF data monthly. EpiData Entry will be performed by the statistical professionals of the Drug Clinical Evaluation Research Center of Central South University. Two personnel will enter the data into the database. The statisticians will check the original data and promptly issue a query to the investigator after identifying problems, then provide data to the recorder for modification. Data management and statistics will be performed after correction.

### Sample size calculation

At present, there is no clinical research report on TCM treatment for WML of CSVD. According to our previous case-control study, Fazekas grades of WML are an independent risk predictor of stroke (*p* < 0.01). With WML levels of 0–3 increasing step by step, delayed memory, spatial, and executive dysfunction appear at WML level 2, specifically at WML level 3. The mean ± standard deviation (SD) of Fazekas scores of HT-CSVD patients was 2.29 ± 1.26 (Zhou et al., 2022). After 6 months of NTF treatment, the mean ± SD of Fazekas scores is 2.29 ± 1.26 for the intervention group and 2.8 ± 1.3 for the control group (since CSVD is a progressive brain disease, we speculate that Fazekas grades of control groups will increase at least 0.5 points after 6 months without intervention). Considering α 10% significance level, the Z-value is based on the Z-value table for two-tailed distribution of 1.65. The β-value is determined at 0.20, and the Z-value is based on the Z-value table for a one-tailed distribution of 0.85. When the dropout rate is 20%, the sample size is calculated using an estimation formula for comparison of the mean values of two independent samples as follow:

$$N=\frac{{\left(Z_{\alpha /2}+Z_{\beta }\right)}^{2}*{\left(1+1/k\right)\;\sigma }^{2}}{\delta^{2}}=\frac{{\left(1.65+0.85\right)}^{2}*2*\left({1.26^{2}+1.3}^{2}\right)/2}{0.5^{2}}=81$$

Where N is the sample size of each group; Z_a_ is the table of Z-value for two-tailed distribution; Z_β_ is the table of Z-value for one-tailed distribution; σ is the standard deviation; δ represents the difference. The number of cases that may drop out is added; thus, 388 subjects will be required.

### Statistical analysis

All statistical analyses will be performed using the SPSS 22.0 statistical software. Descriptive statistics will be used for baseline characteristics data. Quantitative variables (numerical variables) will be analyzed by the normality test (such as *Shapiro-Wilk*), and normal distribution numerical variables will be compared using two independent *t*-tests or analysis of variance (*ANOVA*) and other potential confounding variables (such as multicenter characteristics) as covariates. Numerical variables will be summarized with mean ± SD. Non-parametric tests (*Wilcoxon rank-sum* test, *Kruskal–Wallis* test) and M (P_25_, P_75_) will be used to describe the non-normal distribution. Qualitative variables (categorical variables) are represented by the number of cases and the composition ratio; comparison among groups will be performed using the chi-square test or Fish precision probability. Non-parametric test or analysis of covariance will be used to compare the difference in therapeutic indexes. The efficacy analysis adopts the intention to treat (ITT) analysis and the per-protocol set (PPS). Subgroup analysis will be performed regardless of whether the treatment significant effect the main outcome. Safety analysis will be performed by analyzing the incidence of intergroup AEs and abnormal laboratory examination results before and after treatment.

The full analysis set (FAS) will be conducted for the therapeutic indexes of all subjects included in the study. Statistical analysis in accordance with PPS will be conducted for the therapeutic indexes of subjects completing the drug intervention for 6 months. FAS uses the last-observed-carried-forward method (LOCF) to adjust the missing data and complete ITT analysis. The safety analysis will be performed using the safety analysis set (SS). All statistical tests will be two-sided. *p* ≤ 0.1 means there is a statistical difference, *p* ≤ 0.05 represents a significant statistical difference.

## Discussion

CSVD is the most prevalent, chronic and progressive cerebrovascular disease. The disease caused by various factors including arteriosclerosis and inflammation (Chojdak-Łukasiewicz et al., 2021). A previous meta-analysis revealed that HT, hyperlipidemia, diabetes, and smoking are the risk factors for CSVD (Wang et al., 2021). Moreover, the effects of these risk factors are accelerated by aging (Wang et al., 2021). Therefore, this calls for the development of measures for the early management of these factors, particularly HT. The international STRIVE (Wardlaw et al., 2013) and the Chinese consensus on the diagnosis and therapy of CSVD (Huang, 2015; Hu et al., 2021) released a diagnosis and treatment guidelines for its clinical management. Since then, research on the pathogenesis and treatment of HT-CSVD has gradually attracted attention. The currently used symptomatic and supportive treatment schemes of HT-CSVD involve the management of blood pressure variation (Chen et al., 2019; Zhang et al., 2019), thrombolysis (Powers et al., 2019), antiplatelet therapy (Kim et al., 2020), lipid reduction (Xiong et al., 2014) and others. The above schemes help prevent bleeding and coagulation, as well as suppress stroke and cognitive impairment. Nevertheless, the efficacy of thrombolysis, antiplatelet, and statin drugs is debatable due to the lack of adequate clinical research evidence and the risk of bleeding (Cannistraro et al., 2019). Furthermore, the clinical management of HT-CSVD patients faces many problems and challenges owing to its complex pathogenesis, pathology, and clinical manifestations (Liu et al., 2018). In this regard, identifying effective multi-target Chinese medicines remains a critical research direction of HT-CSVD.

Based on TCM theories and clinical evidence, our research team developed NTF as a new Chinese medicine formula for treating cerebral vascular diseases. We performed a series of investigations to evaluate the pharmacology, efficacy, and safety of NTF for more than 20 years. Studies indicate that NTF and its extracts minimize the cerebral infarct size and improve neurological function by inhibiting inflammation, oxidative stress, and apoptosis (Liao et al., 2015; Lan et al., 2020; Yang et al., 2021; Zhao et al., 2021). Considering the shared pathogenesis pathways between HT-CSVD and ischemic stroke, and if HT-CSVD is not treated in time, it can gradually progress to stroke. Therefore, we speculate that NTF capsules may also be effective against HT-CSVD. Recruitment of participants commenced in July 2020, and the trial is expected to be completed in December 2022. The findings from this study will reassess the effecacy and safety of NTF capsules through a multi-center, double-blind RCT. Moreover, the results of this study will guide the clinical treatment of HT-CSVD and the prevention of other conditions, including stroke and other cerebral macrovascular diseases.

Noteworthy, this trial is still an exploratory study, hence the sample size and clinical research sub-centers may be insufficient. Also, improving participant compliance with medication is a critical issue and worthy of attention, considering that they are elderly with basic diseases. Of importance is that the intervention period of this study is relatively short, and the long-term efficacy and safety will be determined in follow-up studies. Therefore, additional multicenter-clinical trials with large sample sizes and long intervention periods are essential for comprehensive research.

## Conclusion

In summary, this protocol will provide in-depth study information according to the SPIRIT-TCM extension statement. By strictly implementing the trial protocol, this study will generate high-quality evidence on the effecacy and safety of NTF capsules in treating HT-CSVD with QDBS syndrome. Moreover, the results may also help identify the potential mechanism of NTF capsules in treating HT-CSVD with QDBS syndrome. Nonetheless, more clinical trials with large sample sizes and long intervention periods will be required in the future to validate the findings of this trial.

## Abbreviations

ACEI, angiotensin converting enzyme inhibitors; AEs, adverse events; AHD, anti-hypertensive drugs; Ang II, angiotensin II; ANOVA, analysis of variance; ARB, angiotensin receptor blockers; BBB, blood-brain barrier; BMI, body mass index; BP, blood pressure; CCB, calcium channel blockers; CMB, cerebral microbleeds; COX, cyclooxygenase; CRF, case report form; CRA, clinical research associate; CRC, clinical research coordinator; CT, computed tomography; dVRS, dilated virchow-robin spaces; DWI, diffusion weighted imaging; ECG, electrocardiogram; FAS, full analysis set; FLAIR, fluid-attenuated inversion recovery; FPG, fasting plasma glucose; GCP, good clinical practice; GMP, good manufacturing practice; HT-CSVD, hypertensive cerebral small vessel disease; HAMD, Hamilton depression; HbA1c, glycosylated hemoglobin A1c; HT, hypertension; HPLC, high-performance liquid chromatography; ICF, informed consent form; ICH, intracerebral hemorrhage; LI, lacunar infarction; IP, investigational products; LOCF, last-observed-carried-forward method; ITT, intention to treat; MMSE, mini-mental state examination; MRI, magnetic resonance imaging; NOX, NADPH oxidase; NTF, Naotaifang; PPS, per-protocol set; QDBS, Qi deficiency and blood stasis; RAS, renin-angiotensin system; RCT, randomized controlled trial; ROS, reactive oxygen species; RSSI, recent small subcortical infarct; SD, standard deviation; SPIRIT-TCM, Recommendations for Interventional Trials-Traditional Chinese Medicine; SS, safety analysis set; STRIVE, STandards for ReportIng Vascular changes on nEuroimaging; SWI, susceptibility weighted imaging; TCM, traditional Chinese medicine; TLC, thin layer chromatography; WMH, white matter hyperintensity; WML, white matter lesion.

## References

[B1] Amarenco P. Benavente O. Goldstein L. B. Callahan A. 3rd. Sillesen H. Hennerici M. G. (2009). Results of the stroke prevention by aggressive reduction in cholesterol levels (SPARCL) trial by stroke subtypes. Stroke 40 (4), 1405–1409. 10.1161/STROKEAHA.108.534107 19228842

[B2] Bi C. Li P. L. Liao Y. Rao H. Y. Li P. B. Yi J. (2019). Pharmacodynamic effects of dan-hong injection in rats with blood stasis syndrome. Biomed. Pharmacother. 118, 109187. 10.1016/j.biopha.2019.109187 31302425

[B3] Cannistraro R. J. Badi M. Eidelman B. H. Dickson D. W. Middlebrooks E. H. Meschia J. F. (2019). CNS small vessel disease: A clinical review. Neurology 92 (24), 1146–1156. 10.1212/WNL.0000000000007654 31142635 PMC6598791

[B4] Chen X. Zhu Y. Geng S. Li Q. Jiang H. (2019). Association of blood pressure variability and intima-media thickness with white matter hyperintensities in hypertensive patients. Front. Aging Neurosci. 11, 192. 10.3389/fnagi.2019.00192 31447663 PMC6691147

[B5] Cheng C. W. Wu T. X. Shang H. C. Li Y. P. Altman D. G. Moher D. (2017). CONSORT extension for Chinese herbal medicine formulas 2017: Recommendations, explanation, and elaboration. Ann. Intern. Med. 167 (2), 112–121. 10.7326/M16-2977 28654980

[B6] Chinese Pharmacopoeia Commission (2015). “The Pharmacopoeia of people’s republic of China (2015 volume IV),” in Guidelines for stability testing of raw materials medicine and preparations (Beijing: China Medical Science Press), 355–356.

[B7] Chojdak-Łukasiewicz J. Dziadkowiak E. Zimny A. Paradowski B. (2021). Cerebral small vessel disease: A review. Adv. Clin. Exp. Med. 30 (3), 349–356. 10.17219/acem/131216 33768739

[B8] Dai L. Cheng C. W. Tian R. Zhong L. L. Li Y. P. Lyu A. P. (2019). Standard protocol items for clinical trials with traditional Chinese medicine 2018: Recommendations, explanation and elaboration (SPIRIT-TCM extension 2018). Chin. J. Integr. Med. 25 (1), 71–79. 10.1007/s11655-018-2999-x 30484022

[B9] Doubal F. N. MacLullich A. M. Ferguson K. J. Dennis M. S. Wardlaw J. M. (2010). Enlarged perivascular spaces on MRI are a feature of cerebral small vessel disease. Stroke 41 (3), 450–454. 10.1161/STROKEAHA.109.564914 20056930

[B10] Fazekas F. Chawluk J. B. Alavi A. Hurtig H. I. Zimmerman R. A. (1987). MR signal abnormalities at 1.5 T in alzheimer's dementia and normal aging. AJR. Am. J. Roentgenol. 149 (2), 351–356. 10.2214/ajr.149.2.351 3496763

[B11] Folstein M. F. Folstein S. E. McHugh P. R. (1975). Mini-mental state". A practical method for grading the cognitive state of patients for the clinician. J. Psychiatr. Res. 12 (3), 189–198. 10.1016/0022-3956(75)90026-6 1202204

[B12] He Y. H. Hao X. Y. Ge J. W. (2001). Clinical studies on Naotaifang in treating patients with cerebral infarction with deficiency of qi and blood stasis in TCM. Zhong Guo Zhong Yi Ji Zheng 10 (6), 319–320, 334.

[B13] Hu W. L. Yang L. Li H. T. Huang Y. H. (2021). The Chinese consensus on diagnosis and therapy of cerebral small vessel disease 2021. Chin. J. Stroke 16 (7), 716–726. 10.3969/j.issn.1673-5765.2021.07.013

[B14] Huang Y. N. (2015). Chinese consensus on diagnosis and therapy of cerebral small vessel disease. Chin. J. Neurology 48 (10), 838–844. 10.3760/cma.j.issn.1006-7876.2015.10.004

[B15] Kaul M. Rubinstein I. (2021). Population-based magnetic resonance imaging: Earlier detection of hypertensive cerebral small vessel disease? Hypertension 78 (2), 540–542. 10.1161/HYPERTENSIONAHA.121.17606 34232679 PMC8284836

[B16] Kim B. J. Kwon S. U. Park J. H. Kim Y. J. Hong K. S. Wong L. K. S. (2020). Cilostazol versus aspirin in ischemic stroke patients with high-risk cerebral hemorrhage: Subgroup analysis of the PICASSO trial. Stroke 51, 931–937. 10.1161/STROKEAHA.119.023855 31856691

[B17] Lan B. Ge J. W. Cheng S. W. Zheng X. L. Liao J. He C. (2020). Extract of Naotaifang, A compound Chinese herbal medicine, protects neuron ferroptosis induced by acute cerebral ischemia in rats. J. Integr. Med. 18 (4), 344–350. 10.1016/j.joim.2020.01.008 32107172

[B18] Li Y. Y. Huang N. Q. Feng F. Li Y. Luo X. M. Tu L. (2020). Icaritin improves memory and learning ability by decreasing BACE-1 expression and the bax/bcl-2 ratio in senescence-accelerated mouse prone 8 (SAMP8) mice. Evid. Based. Complement. Altern. Med. 2020, 8963845. 10.1155/2020/8963845 PMC7345953 32714426

[B19] Liao J. Xia X. Wang G. Z. Shi Y. M. Ge J. W. (2015). Naotaifang extract treatment results in increased ferroportin expression in the Hippocampus of rats subjected to cerebral ischemia. Mol. Med. Rep. 11 (6), 4047–4052. 10.3892/mmr.2015.3309 25672910 PMC4394947

[B20] Lin H. Y. Fang R. Zhang Z. X. Zhou Y. Wang S. S. Jiang Q. L. (2021). Study on the action mechanism of Naotaifang in the prevention and treatment of hypertensive cerebral small vessel disease based on network pharmacology and molecular docking. Mod. Traditional Chin. Med. Materia Medica-World Sci. Technol. 23 (12), 4364–4373. 10.11842/wst.20210807001

[B21] Litak J. Mazurek M. Kulesza B. Szmygin P. Litak J. Kamieniak P. (2020). Cerebral small vessel disease. Int. J. Mol. Sci. 21 (24), 9729. 10.3390/ijms21249729 33419271 PMC7766314

[B22] Liu W. Zhou L. Feng L. Zhang D. Zhang C. Gao Y. (2021). Buqitongluo granule for ischemic stroke, stable Angina pectoris, diabetic peripheral neuropathy with qi deficiency and blood stasis syndrome: Rationale and novel basket design. Front. Pharmacol. 12, 764669. 10.3389/fphar.2021.764669 34733163 PMC8558407

[B23] Liu Y. Dong Y. H. Lyu P. Y. Chen W. H. Li R. (2018). Hypertension-induced cerebral small vessel disease leading to cognitive impairment. Chin. Med. J. 131 (5), 615–619. 10.4103/0366-6999.226069 29483399 PMC5850681

[B24] Lu Y. W. Hao R. J. Wei Y. Y. Yu G. R. (2021). The protective effect of harpagoside on angiotensin ii (ang ii)-induced blood-brain barrier leakage *in vitro* . Phytother. Res. 35 (11), 6241–6254. 10.1002/ptr.7269 34486189

[B25] Powers W. J. Rabinstein A. A. Ackerson T. Adeoye O. M. Bambakidis N. C. Becker K. (2019). Guidelines for the early management of patients with acute ischemic stroke: 2019 update to the 2018 guidelines for the early management of acute ischemic stroke: A guideline for healthcare professionals from the American heart association/American stroke association. Stroke 50, e344–e418. 10.1161/STR.0000000000000211 31662037

[B26] Wang Y. Yang J. H. Wan H. T. He Y. Xu B. Ai C. S. (2021). Efficacy of yangyin yiqi huoxue granule in treatment of ischemic stroke patients with qi-yin deficiency and blood stasis syndrome: A randomized, double-blind, multicenter, phase-2 clinical trial. Chin. J. Integr. Med. 27 (11), 811–818. 10.1007/s11655-021-2857-0 33881715

[B27] Wang Z. Chen Q. Chen J. Yang N. Zheng K. (2021). Risk factors of cerebral small vessel disease: A systematic review and meta-analysis. Med. Baltim. 100 (51), e28229. 10.1097/MD.0000000000028229 PMC8702220 34941088

[B28] Wardlaw J. M. Smith E. E. Biessels G. J. Cordonnier C. Fazekas F. Frayne R. (2013). Neuroimaging standards for research into small vessel disease and its contribution to ageing and neurodegeneration. Lancet. Neurol. 12 (8), 822–838. 10.1016/S1474-4422(13)70124-8 23867200 PMC3714437

[B29] Writing Group of Chinese Guidelines for the Management of Hypertension (2019). Chinese hypertension league., Chinese society of cardiology, Chinese medical doctor association hypertension committee., hypertension branch of China international exchange and promotive association for medical and Health care., and hypertension branch of Chinese geriatric medical AssociationChinese guidelines for the management of hypertension. Chin. J. Cardiovasc. Med. 24 (1), 24–56. 10.3969/j.issn.1007-5410.2019.01.002

[B30] Xiong Y. Wong A. Cavalieri M. Schmidt R. Chu W. W. Liu X. (2014). Prestroke statins, progression of white matter hyperintensities, and cognitive decline in stroke patients with confluent white matter hyperintensities. Neurotherapeutics 11 (3), 606–611. 10.1007/s13311-014-0270-5 24692001 PMC4121460

[B31] Xu D. Ai Q. Chen X. Wang Z. Wei H. Zhou L. (2022). Metabonomics study on Naotaifang extract alleviating neuronal apoptosis after cerebral ischemia-reperfusion injury. Evid. Based. Complement. Altern. Med. 2022, 2112433. 10.1155/2022/2112433 PMC8938065 35321499

[B32] Yang T. Chen X. Mei Z. Liu X. Feng Z. Liao J. (2021). An integrated analysis of network pharmacology and experimental validation to reveal the mechanism of Chinese medicine formula Naotaifang in treating cerebral ischemia-reperfusion injury. Drug Des. devel. Ther. 15, 3783–3808. 10.2147/DDDT.S328837 PMC8434864 34522084

[B33] Zhang H. Cui Y. Zhao Y. Dong Y. Wang J. Duan D. (2019). Association of circadian rhythm of blood pressure and cerebral small vessel disease in community-based elderly population. J. Gerontol. A Biol. Sci. Med. Sci. 74 (8), 1322–1330. 10.1093/gerona/gly212 30252020

[B34] Zhang L. Tang X. Li Y. Zhu J. Ding D. Zhou Y. (2022). Total magnetic resonance imaging of cerebral small vessel disease burden predicts dysphagia in patients with A single recent small subcortical infarct. BMC Neurol. 22 (1), 1–39. 10.1186/s12883-021-02518-9 34979972 PMC8722168

[B35] Zhang W. J. Su W. W. Lin Q. W. He Y. Yan Z. H. Wang Y. G. (2020). Protective effects of naoxintong capsule alone and in combination with ticagrelor and atorvastatin in rats with qi deficiency and blood stasis syndrome. Pharm. Biol. 58 (1), 1006–1022. 10.1080/13880209.2020.1821066 32985308 PMC7534269

[B36] Zhao D. Yi Y. He Q. Wang S. Yang K. Ge J. (2021). Exploring the regulatory mechanism of nao tai fang on vascular dementia's biological network based on cheminformatics and transcriptomics strategy. J. Ethnopharmacol. 274, 114065. 10.1016/j.jep.2021.114065 33771644

[B37] Zhen X. Y. and Guiding principles for clinical study of new Chinese medicines (2002). The guiding principles of clinical research on the treatment of diarrhea with new Chinese medicine, 75. Beijing: China Medical Science Press, 102.

[B38] Zhou H. Gao F. Yang X. Lin T. Li Z. Wang Q. (2022). Endothelial BACE1 impairs cerebral small vessels via tight junctions and eNOS. Circ. Res. 130 (9), 1321–1341. 10.1161/CIRCRESAHA.121.320183 35382554

[B39] Zhou Y. Fang R. Liu X. Xie L. Wang W. Tong J. (2022). Correlation analysis of blood lipid, coagulation function and total bilirubin with hypertension combined with white matter lesions. J. Hunan Univ. Chin. Med. 42 (5), 794–799. 10.3969/j.issn.1674070X.2022.05.017

